# Infantile‐onset CMT2D/dSMA‐V in a Chinese family with parental germline mosaicism for a novel mutation in the *GARS1* gene

**DOI:** 10.1002/mgg3.1846

**Published:** 2021-12-12

**Authors:** Yufeng Huang, Bo Bi, Peiwei Zhao, Ting Yu, Sukun Luo, Li Tan, Zhisheng Liu, Jie Liu, Xuelian He

**Affiliations:** ^1^ Precision Medical Center Wuhan Children's Hospital (Wuhan Maternal and Child Healthcare Hospital) Tongji Medical College Huazhong University of Science & Technology Wuhan China; ^2^ Department of Rehabilitation Wuhan Children's Hospital (Wuhan Maternal and Child Healthcare Hospital) Tongji Medical College Huazhong University of Science & Technology Wuhan China; ^3^ Department of Gastroenterology Wuhan Children's Hospital (Wuhan Maternal and Child Healthcare Hospital) Tongji Medical College Huazhong University of Science & Technology Wuhan China

**Keywords:** CMT2D/dSMA‐V, exome sequencing, *GARS1*, infant onset, mosaic

## Abstract

**Background and Aims:**

Both Charcot‐Marie‐Tooth disease type 2D (CMT2D) and distal spinal muscular atrophy type V (dSMA‐V) are GARS1 disease phenotypes involving axonal peripheral neuropathy. Patients often develop clinical symptoms in their teens. Herein, we reported a Chinese family with infantile‐onset CMT2D/dSMA‐V.

**Methods:**

Clinical evaluation and laboratory examination were performed in our proband, the older sister from this family, and trio exome sequencing (ES) was conducted on the proband and her parents, followed by Sanger sequencing.

**Results:**

A novel *GARS1* mutation (c.997G>C, p.E333Q; NM_002047) was identified in this patient and her younger sister but not in her parents; thus, it is presumed that this mutation is inherited from a germline mosaic parent. The younger sister began to exhibit weakness of her hands and feet at the age of 1 year old.

**Conclusion:**

This is the first report of infantile CMT2D/dSMA‐V in China. Our study increases the number of infantile‐onset cases, as well as reported pathogenic variants in the *GARS1* gene, and highlights the important role of exome sequencing in the clinical diagnosis of disease and enabling subsequent prenatal diagnosis. Our study reminds us to consider the possibility of parent germline mosaicism in the subsequent prenatal genetic diagnosis when identifying a de novo variant.

## Graphical abstract

This is the first report of infantile CMT2D/dSMA‐V in China. It is presumed that this mutation is inherited from a germline mosaic parent. Our study reminds us to consider the possibility of parent germline mosaicism in the subsequent prenatal genetic diagnosis when identifying a de novo variant.

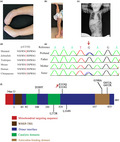

## 1 INTRODUCTION

Charcot‐Marie‐Tooth disease type 2D (CMT2D, OMIM 601472) is axonal peripheral motor and sensory neuropathy caused by mutations in the glycyl‐tRNA synthetase 1 (*GARS1*) gene (OMIM 600287; Ionasescu et al., 1996). *GARS* is also the pathogenic gene causing distal spinal muscular atrophy type V (dSMA‐V, OMIM 600794; Antonellis et al., 2003). The phenotypes of CMT2D and dSMA‐V are similar, and the main difference is sensory deficits in the distal limbs in the former (Antonellis et al., 2003).

There are 37 genes encoding tRNA synthetases in humans, and tRNA synthetases are essential for protein synthesis by charging tRNAs with their cognate amino acids. Among these 37 genes, *GARS1* is the first gene identified to be implicated in CMT (Antonellis et al., 2003). The *GARS1* gene is located on chromosome 7q14, contains 17 exons, and encodes two forms of tRNA synthetase: mitochondrial (739 aa) or cytoplasmic (685 aa), and they have three major functional domains: a highly conserved WHEP‐TRS (residues 13–63), catalytic core (residues 92–168 and 241–324), and an anticodon‐binding domain (residues 557–655), and three dimer interface regions.

CMT2D/dSMA‐V typically presents with progressive muscle weakness and atrophy with adolescent or early adult onset (Antonellis et al., 2003); however, a few infantile or early childhood onset cases have been reported (Chae et al., 2015; Chung et al., 2018; Eskuri et al., 2012; James et al., 2006; Liao et al., 2015; Markovitz et al., 2020). Here we report, for the first time, a Chinese family with infantile onset of CMT2D/dSMA‐V caused by *GARS1* mutation (c.997G>C; NM_002047) probably inherited from mosaic parental germline mutation. Our study enriches the knowledge of mutation and phenotype spectrum in CMT2D/dSMA‐V patients and highlighted the important role of whole‐exome sequencing in clinical genetic diagnosis and subsequent prenatal diagnosis.

## 2 MATERIALS AND METHODS

### 2.1 Subjects

A 7‐month‐old girl was admitted with a suspected diagnosis of peripheral neuropathy. Peripheral blood samples of the girl and her normal parent and younger sister were collected. Written informed consent was obtained from the girl's parent, and the study was approved by the ethics committee of Wuhan Children's Hospital (approval number 2019011).

### 2.2 Exome sequencing

ES and subsequent variant analysis were carried out in the medical testing laboratory (Chigene Lab). Genomic DNA was isolated from peripheral blood, and trio‐ES was carried out on the proband and her parents. A potentially pathogenic *GARS1* variant was identified. The clinical effects of variants identified were classified into five categories according to the American College of Medical Genetics and Genomics and the Association for Molecular Pathology (Kalia et al., 2016). PolyPhen‐2 (http://genetics.bwh.harvard.edu/pph/), SIFT (http://www. Blocks.fhcrc.org/sift/SIFT.html), and MutationTaster (http://www.mutationtaster.org/) were performed to predict the possible effects of the variants identified as mentioned earlier (Tan et al., 2021). PCR and Sanger sequencing were carried out to validate the mutation identified by ES using self‐designed primers. The genomic DNA from the younger sister was used to verify the mutation, c.997G>C (p.E333Q), identified in the proband. To examine the conservation of the amino acid affected by the mutation, Clustal Omega was used to align the sequences of GARS protein from zebrafish, T. trubripes, mice, humans, and chimpanzees.

## 3 RESULTS

### 3.1 Clinical information

**FIGURE 1:**
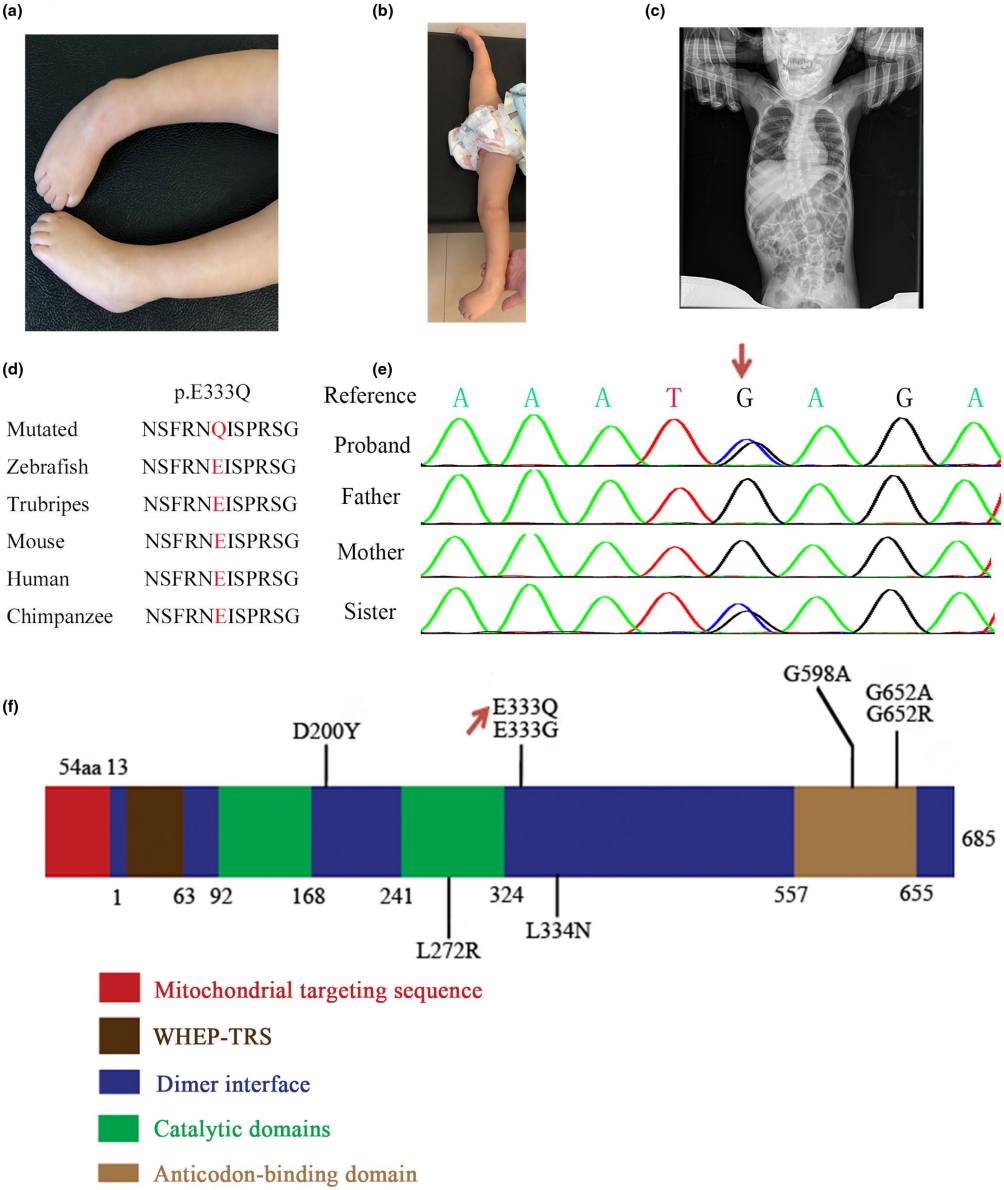
Clinical features of proband, genetic findings from family, and schematic diagrams of GARS1. (a,b) The clinical manifestations of our patient. (c) A “S” shape of lateral of curvature of the spine shown by X‐ray. (d) Sanger sequencing to confirm the *GARS1* c.997G>C mutation in the girl. The mutation is marked by a black arrow. (e) Conservation analysis of the mutation (p.E333Q) in *GARS1* across species. (f) Schematic diagrams showing structure of GARS1 protein, mutation identified our patient marked by a red arrow

The proband was the first child of healthy, nonconsanguineous parents without inherited diseases, and she was born at 40 weeks of gestation after an uneventful pregnancy. She had no obvious evidence to indicate any abnormality in movement, breath, and feeding before 6 months old. At the age of 7 months, her parents noticed that she was unable to sit independently, had claw hands, “floppy” feet, and had notable hypotonia in her extremities (Figure 1a). At the age of 1 year old, she was hospitalized due to progressive weakness in distal limbs, hunched back, and weak cry. She had significant generalized hypotonia and difficulty to overcome gravity strength in her lower extremities, and she had excessive abduction of the hip with 180 degrees of abduction angle (Figure 1b). Nerve conduction study with electromyography (EMG) was performed and revealed a motor peripheral neuropathy, as the muscle action potentials were decreased in all tested nerves, including left median and ulnar, bilateral tibial and femoral, right peroneal nerves, while the sensory nerve action potentials were normal in the median and ulnar nerves. Multiple fibrillation potentials and positive sharp waves were seen in muscle of left extensor digitus totalis, medial head of gastrocnemius, tibialis anterior, and femoris internus. Based on the results of the EMG, the patient was diagnosed with axonal neuropathy. There was no obvious clinical evidence to indicate a superficial sense to be impaired. X‐ray revealed thinner diaphysis in the femur, tibia, and fibula compared with children of the same age, and an “S” shape of lateral curvature of the spine (Figure 1c). At the age of 1 year and 9 months, she developed severe scoliosis, and her hands were losing the ability to grab and hold things. At the age of 2 years old, she progressed to respiratory failure, but the proposal of mechanical ventilation was refused by her guardian, and she died at the age of 2 years and 3 months. Her younger sister began to develop low muscle strength in limbs at 1 year old, and the genetic test revealed the same *GARS1* mutation in her peripheral blood, probably inherited from germline mosaic parent.

### 3.2 Trio‐ES identified GARS as a candidate pathogenic gene

By using trio‐ES, after the filtering steps, a heterozygous missense variant, c.997G>C (p.E333Q), in exon 8 of the *GARS1* gene (NM_002047) was identified in the proband but not in her parents. Further Sanger sequencing confirmed this result (Figure 1d). This missense variant is predicted to be pathogenic by ACMG guidelines and bioinformatics tools as described above and the encoded amino acid residue is conserved among different species (Figure 1e). The c. 997G>C variant has not been reported previously and is not present in any public genomic variants database, including Human Genome Mutation Database (HGMD, http://www.hgmd.cf.ac.uk/ac/index.php), 1000 Genomes (https://www.internationalgenome.org/), ESP6500 (https://evs.gs.washington.edu/EVS/), ExAC Browser (http://exac.broadinstitute.org/), and COSMIC (https://cancer.sanger.ac.uk/cosmic/). However, it is next to a reported pathogenic variant affecting c.998, which is part of the same codon for the 333rd amino acid residue (Chae et al., 2015). All these findings suggest c.997G>C in *GARS1* as a pathogenic mutation. Although this variant is a novel one, Sanger sequencing was performed to test her younger sister's peripheral blood, and sequencing result showed that this variant is present, indicating germline mosaic in one of their parents.

## 4 DISCUSSION

Pathogenic variants in *GARS1* are associated with CMT2D or dSMA‐V, depending on whether sensory nerves are affected, and the sensory deficits present in the former. The typical presentation is a gradually progressive weakness in limbs, and the age of onset varies from second to fourth decades of life. A few cases were reported to develop symptoms during infancy and early childhood. In this study, we reported two siblings from a Chinese family with CMT2D/dSMA‐V caused by a *GARS1* mutation (c.997G>C, p.E333Q) inherited from a germline mosaic parent.

To date, a total of 12 patients have been reported to present clinical symptoms of CMT2D/dSMA‐V at infancy or early childhood, caused by seven missense mutations, c.598G>T (p.D200Y), c.815T>G (p.L272R), c.998A>G (p.E333G), c.1001T>A (p.L334N), c.2313G>C (p.G598A), c.1954G>C (p.G652R), and c.1955G>C (p.G652A), respectively (Chae et al., 2015; Chung et al., 2018; Eskuri et al., 2012; James et al., 2006; Liao et al., 2015; Markovitz et al., 2020). As shown in Table 1 and Figure 1f, among these variants, only the variant (p.L272R) lies on catalytic domain, three mutations, p.G652R, p.G652A, and p.G598A on anticodon‐binding domains, and the other three (p.D200Y, p.E333G, and p.L334N) on dimer interface regions (Figure 1f). Interestingly, the mutation (c.997G>C, p.E333Q) in our patients occurs at the same amino acid residue reported in a floppy infant from Korea^6^ and is adjacent to the 334th residue which was reported to be mutated in two unrelated Hispanic patients. Given the rarity of *GARS1* mutations and the low frequency of *GARS1* mutations in patients with CMT2D, the 333rd and 334th residues may represent hot spots of mutation in the *GARS1* gene to be associated with infantile or early childhood onset CMT2D/dSMA‐V.

**TABLE 1:** Clinical features of infant onset patients from reference and our study and molecular features of mutations in the *GARS1* gene

	Patient 1	Patient 2	Patients 3 and 4 (twins)	Patient 5	Patient 6	Patient 7	Patient 8	Patient 9	Patient 10	Patient 11	Patient 12
Author		James et al	Eskuri et al	Liao et al	Chae et al	Chung et al			Markovitz et al		
Year of publication		2006	2012	2015	2015	2018			2020		
Country or region	China	Landon	USA	Taiwan	Korea	Nigeria			Hispania		
Sex	F	F	F	F	M	F	F	M	M	M	M
Age of onset	7 months	6 months	6 months	3 months	Since birth	10 months	9 months	7 months	9 weeks	6 weeks	Since birth
First symptoms	Varus and “floppy” feet, hands hold things unstably	“Floppy” feet	No spontaneous movement of feet or toes	Pneumonia with respiratory failure	Severe hypotonia	Acute respiratory failure	Delayed gross motor milestones	Acute respiratory failure	Respiratory distress, poor feeding, and muscle weakness	Repeated events of respiratory failure	Inspiratory stridor and hypotonia
Inheritance	Inherited	De novo	De novo	De novo	De novo	From chimeric mother			De novo		
Mutation	c. 997G>C (p.E333Q)	c.2313G>C (p.G598A)	c.1955G>C (p.G652A)	c.598G>T (p.D200Y)	c.998A>G (p.E333G)	c.815T>G (p.L272R)			c.1001T>A (p.L334N)	c.1001T>A (p.L334N)	c.1954G>C (p.G652R)
Domain mutation locates in	Dimer interface	Anticodon‐binding domain	Anticodon‐binding domain	Dimer interface	Dimer interface	Catalytic domains			Dimer interface	Dimer interface	Anticodon‐binding domain
Methods of mutation analysis	Trio exome sequencing	PCR, DHPLC, Sanger sequencing	PCR, Sanger sequencing	NGS (panel)	NGS (panel)	Exome sequencing			Trio exome sequencing	NGS (panel)	Trio exome sequencing
Axial and truncal (proximal) weakness	ND	−	ND	ND	ND	+	+	+	+	+	+
Hands/feet extremities (distal) weakness	+	+	+	+	ND	+	+	+	+	+	+
Pes Cavus	‐	ND	ND	Pes planus at 9 years old	ND	−	−	−	−	+	−
Distal axonal neuropathy with UE > LE	No, LE symptoms >UE symptoms	No, LE symptoms >UE symptoms	UE symptoms = LE symptoms	UE symptoms = LE symptoms	ND	ND	ND	ND	No, LE symptoms >UE		
symptoms	No, LE symptoms >UE symptoms	No, LE symptoms >UE symptoms									
Sensory impairment	ND	−	−	+	ND	−	−	ND	ND	+	−
Femorotibial angle	180°	ND	ND	−	ND	ND	ND	ND	ND	ND	ND
Hyporeflexia	+	+	+	+	ND	+	+	+	+	+	+
Hypotonia	+	+	+	+	+	+	+	+	+	+	+
Scoliosis	+	+	No in Twin A; Yes in Twin B	−	ND	−	−	−	−	+	−
Independent ambulation	−	−	−	−	ND	−	−	−	−	−	−
Respiratory problems	Cough, pneumonia, and atelectasis	Weak cough, using accessory muscles of respiration	Inspiratory stridor in one	Pneumonia with respiratory failure was cured with treatment	ND	Acute respiratory failure	−	Acute hypercapnic respiratory failure	Stridor, weak cry, elevated right diaphragm, and ehronic ventilation	Stridor, weak cry, elevated right diaphragm, and ehronic ventilation	Stridor, weak cry, Poor diaphragm movement, and ehronic ventilation
Outcome	Die	ND	ND	ND	ND	ND	ND	ND	ND	ND	ND

The first reported case of infantile‐onset dSMA‐V was from Landon in 2006, and the patient began to present floppy feet at the age of 6 months and never achieved independent walking, and presented marked distal weakness and wasting in the legs, hyperlordosis, and scoliosis by 7 years (James et al., 2006). The G598A mutation in the anticodon‐binding domain of GARS was identified by denaturing high‐performance liquid chromatography (DHPLC) followed by PCR and Sanger sequencing (James et al., 2006). In 2012, Eskuri et al. reported monozygotic twin girls from the United State with a severe, infantile‐onset CMT2D/dSMA‐V (Eskuri et al., 2012), similar to the patient reported by James et al. (2006), and a mutation (G652A) within the anticodon‐binding domain of GARS1 was found by direct Sanger sequencing (Eskuri et al., 2012). Afterward, eight individuals with infantile‐onset CMT/dSMA‐V were reported from Taiwan, Korea, and the USA, and the genetic tests were performed by next‐generation sequencing (NGS), including panel, ES, or trio‐ES (Chae et al., 2015; Chung et al., 2018; Eskuri et al., 2012; James et al., 2006; Liao et al., 2015; Markovitz et al., 2020). All these mutations identified in these patients were de novo, except three siblings from a Nigerian descent family in the USA, whose variant (c.815T>G, p.L272R) was inherited from their healthy mother with low‐level (10%–20%) mosaicism (Chung et al., 2018).

Given the rarity of the infantile or early childhood onset CMT2D/dSMA‐V caused by *GARS1*, most commercial gene panels for early onset CMT or SMA may not contain this gene, therefore, the diagnosis of *GARS1*‐related disease may be missed or delayed. As the cost of high‐throughput sequencing has fallen, ES has the advantage to investigate all coding genes not only target genes and improves the genetic diagnostic rate for rare diseases. Most genetic diagnoses in these patients with early onset CMT2D/dSMA‐V were made based on ES or trio‐ES in literatures (Table 1).

In this study, we found a variant, c.997G>C (p.E333Q), in the dimer interface of GARS1 by ES in our patient with infant onset of CMT2D/dSMA‐V. This amino acid encoded by the variants located in the same amino acid residue where adjacent base substitution, c.998A>G (p.E333G), had previously been identified as a pathogenic one, and was highly conserved and predicted to be pathogenic by bioinformatics tools, and our patient displayed similar phenotype as those previously reported (Chae et al., 2015; Chung et al., 2018; Eskuri et al., 2012; James et al., 2006; Liao et al., 2015; Markovitz et al., 2020). Since this variant was not found in the peripheral blood of the parents, but in the peripheral blood of her younger sister, we speculated that germline cells were mosaic in one parent. During the follow‐up, we are informed that the younger sister also presented progressive weakness in limbs, distal great than proximal.

Besides adolescent‐onset or infant‐onset neuromuscular disorders, *GARS1* mutations had been reported to be associated with other diseases such as mitochondrial disease, autism spectrum disorder, and a multisystem developmental syndrome that includes severe growth retardation (Mcmillan et al., 2014; Oprescu et al., 2017; Yuen et al., 2015). One patient (P31) reported by Taylor et al. was suspected to have a mitochondrial disease after birth, with muscle and heart involved, and dead in 1 month old, with multiple respiratory chain complex defects. This patient was found to be homozygous for the variant (c.2065C>T, p.Arg689Cys) in *GARS1*, and his parents were consanguineous (Taylor et al., 2014). The patient reported by Oprescu presented a severe, multisystem, developmental phenotype with growth retardation and is compound heterozygous for one frameshift (p.Glu83Ilefs*6) and one missense (p.Arg310Gln) *GARS* variant, inherited from his parent, respectively (Oprescu et al., 2017). None of these two patients’ parents displayed signs of CMT2D or dSMA‐V, which could indicate that haploinsufficiency is not a likely mechanism of *GARS*‐associated CMT2D/dSMA‐V. All these cases address the phenotypic heterogeneity and complexity of the molecular mechanism of the variants in *GARS1* gene.

In conclusion, our study reported a new variant in the *GARS1* gene leading to infant onset CMT2D/dSMA‐V, underlying the important role of exome sequencing in clinical diagnosis of disease and enabling subsequent prenatal diagnosis. This mutation identified in our study expands the mutation spectrum of *GARS1* and reminds us to be alert to the possibility of parent germline mosaicism in the subsequent prenatal genetic diagnosis when identifying a de novo variant.

## CONFLICT OF INTEREST

The authors have declared no conflicts of interest.

## AUTHOR CONTRIBUTIONS

Study concepts: XH, YH. Study design: YH, BB, JL. Literature research: BB, JL. Clinical information collection: ZL, YH, LT. Data acquisition: YH, BB, JL. Data analysis/interpretation: PZ, TY, SL. Manuscript preparation: YH, XH. Manuscript editing/revision/review: ZL, JL.
